# Cutaneous Mucormycosis in a Diabetic Patient following Traditional Dressing

**DOI:** 10.1155/2013/894927

**Published:** 2013-07-29

**Authors:** Zahra Ahmadinejad, Hamideh Khazraiyan, Fahime Ghanbari, Bahram Ahmadi, Mohsen Gerami Shoar

**Affiliations:** ^1^Department of Infectious Diseases, Imam Khomeini Hospital, Tehran University of Medical Sciences, Keshavarz Boulevard, Tehran 14195, Iran; ^2^Department of Medical Mycology and Parasitology, Tehran University of Medical Sciences, Tehran 14195, Iran

## Abstract

Cutaneous mucormycosis is a rare manifestation of an aggressive fungal infection. Early diagnosis and treatment are vitally important in improving outcome. We report an unusual case presenting with progressive necrotizing fasciitis due to mucormycosis following trauma and dressing by man-made herbal agents.

## 1. Introduction 

 Mucormycosis is a rare infection. The infection is more common among people with suppressed immune systems, but it can rarely occur in healthy people. Known risk factors for developing mucormycosis are uncontrolled diabetes mellitus, metabolic acidosis, high dose of corticosteroid, prolonged neutropenia, organ transplantation, skin trauma (cuts, scrapes, punctures, or burns), and catheter infection [1–3].

The syndromic manifestations of mucormycosis are recognized by rhinocerebral, pulmonary, gastrointestinal, dermal, or disseminated involvement [1]. Although dermal involvement rarely occurs, the instant diagnosis can lead to reduction of mortality and morbidity [1]. We report an unusual case of skin mucormycosis following trauma and bandaging by man-made herbal agents. 

## 2. Case Report 

A 63-year-old poorly controlled diabetic lady treated with an oral antihyperglycemic agent (Metformin 500 mg/BD) was admitted to our hospital, with complaint of severe pain, swelling, and bruising of the left upper extremity. 

The patient had a history of the wrist strain about four days before admission, in avoidance of falling from a vehicle which resulted in wrist dislocation. The patient had been visited by an alternative practitioner and a wrist reduction was done unprofessionally for her. He bandaged up her wrist with some man-made herbal combination (including egg, flour, and turmeric). 

**Figure 1:**
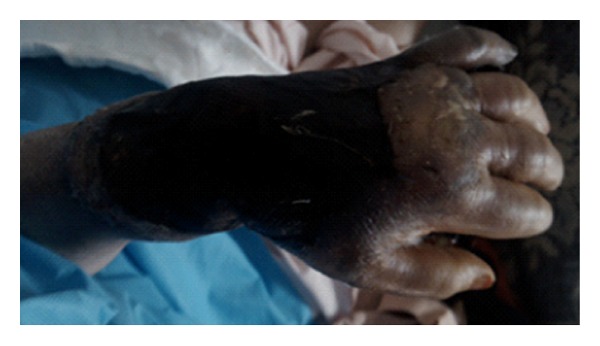
Rapidly progressive skin and deep soft tissue necrosis of the hand and forearm.

On the fourth day after injury, the pain was progressive despite herbal dressing and taking analgesic. Her bandage was unwrapped by her companions and because of a necrotizing region covering from her hand to forearm; she was admitted to Imam Khomeini Hospital (a 1000-bed, referral, tertiary teaching hospital, in Tehran, Iran) in September 2011. On examination, her general condition was fair, but she had a low grade fever (oral temperature = 38 degrees centigrade) and she was distressed by her left-hand pain. Her respiratory, cardiovascular systems and abdomen were within normal limit. Local examination showed hand, wrist, and forearm edema, a black skin discoloration, and cutaneous necrosis (Figure 1). The region was warm and tender without erythema or induration. Also a limitation in wrist range of motion was detected. The radial pulse was palpable, while ulnar pulse was hardly palpable. There was neither crepitation nor fluctuation. 

The patient was treated with Clindamycin and Cefazolin with a diagnosis of cellulitis. The laboratory findings were unremarkable except for an anemia (Hb = 11.4 mg/dL), a mild elevation of erythrocyte sedimentation rate (42 mm/hour), and high levels of C-reactive protein (78 mg/dL).

Ultrasonography showed a collection with a fair septation on the left hand, measured 38 × 42 × 38 (in depth) mm. A collection around the metacarpal-phalangeal joints and thickness in soft tissue and fat echo were also seen. Subcutaneous edema with undetermined boarders was reported too. 

The patient was operated with diagnosis of tenosynovitis. The tendons exploration was done and a small (5 cc) bloody fluid was aspirated. The patient was still febrile after 24 hours and she was suffering from severe tenderness in her fingers and wrist. The Color Doppler Sonography showed normal arterial flow in the brachial vessels and the proximal of radial and ulnar arteries, but a thrombosis was detected in the distal compartment of radial, the middle compartment of ulnar was without arterial flow too. The computed tomography (CT) angiography had a technical problem and the distal vessels of forearm were unobservable.

On the fifth day of admission, antibiotics changed to piperacillin tazobactam (Tazocin) and Vancomycin. Heparin was started for deep vein thrombosis. On the 10th day of admission, considering the expansion of skin necrosis area to the ulnar region of palmer in addition to her whole fifth finger, despite the broad-spectrum antibiotic therapy, conventional amphotericin b was started suspecting cutaneous mucormycosis. 

**Figure 2:**
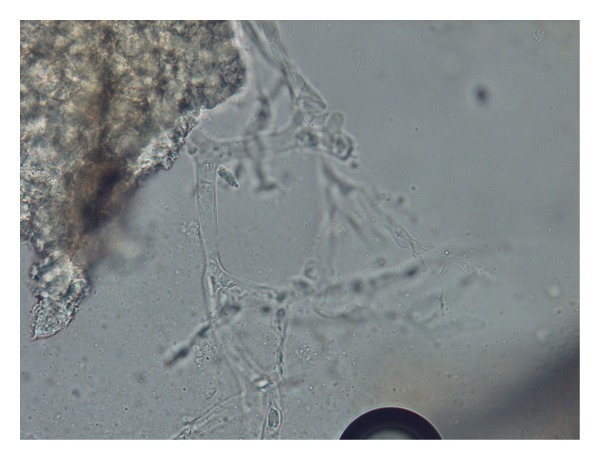
Microscopic features of the causing agent of mucormycosis after treating with 10%KOH showed broad nonseptate hyphae (×400).

**Figure 3:**
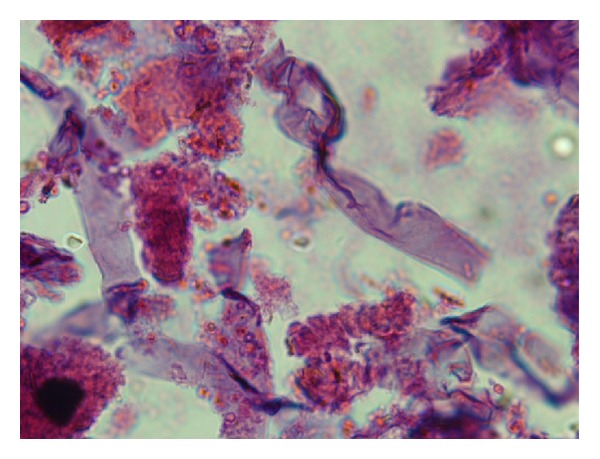
Histopathology showing broad, aseptate, and thin walled fungal hyphae in biopsy specimen (H&E ×400).

**Figure 4:**
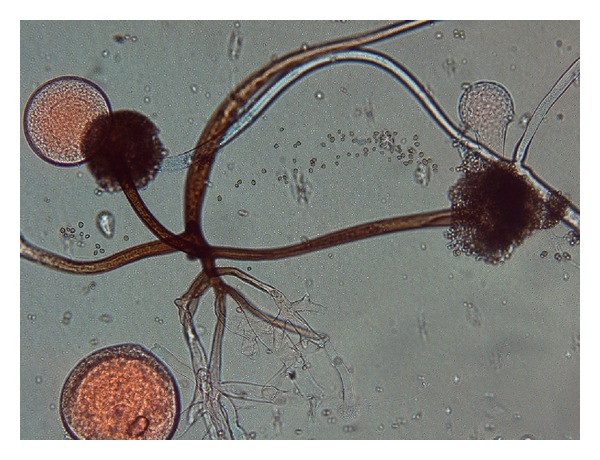
Lactophenol cotton blue (LPCB) mount of growth of *Rhizopus* spp., on Sabouraud's dextrose agar without cycloheximide.

The surgical debridement was repeated and a tissue biopsy was performed to assess for fungal elements. A smear and tissue biopsy showed large, broad, and nonseptate fungal hyphae and the tissue culture was positive for *Rhizopus* species (Figures 2, 3, and 4). 

Finally, the left below elbow amputation was done for the patient on 18 days of admission due to progressive skin necrosis and limb gangrene and failure of medical therapy. Amphotericin b was continued for 9 days. The lady was alive without any problem six months after hospital discharge. 

## 3. Discussion 

Mucormycosis is an invasive fungal infection that could appear during environmental contact and trauma [1, 2]. It is also found in decay matter. Risk factors for mucormycosis are as follows: diabetes and diabetic ketoacidosis, major trauma, high dose of corticosteroid, neutropenia, iron overload, mal-absorption, prematurity status, and drug history of antifungal drugs not effective on zygomycetes, for example, voriconazole and echinocandins [4]. 

Cutaneous mucormycosis is a rare case which is seen in minor trauma cases [2]. In a healthy man, traumatic injury is a known factor for accelerating the process of mucormycosis infection. Furthermore, in an immunosuppressed patient, it can cause the expansion of infection into deeper layer and also produce the disseminated infection [5]. Sporadic cases have been linked with contaminated bandages, needles, and adhesive dressings [4–6]. 

Predisposing factors for developing mucormycosis in our patient were diabetes mellitus, trauma, and contact with herbal combination possibly contaminated by zygomycetes.

A typical clinical presentation of cutaneous mucormycosis is a necrotic scar surrounded by an erythematous and indurated region of skin. However, it could be presented in different forms including tinea corporis like lesion (a superficial lesion with squamous borders), erythema multiform like lesion, and lesions with a cotton-like appearance [1]. In an immunosuppressed patient, a small erythematous macula, even tiny and nonspecific, could be the primary sign of a disseminated mucormycosis [1]. Clinical expertise and a high index of suspicion are two required factors in timely diagnosis of a patient with cutaneous mucormycosis. Although our patient had a necrotic scar, nonspecific manifestations, low clinical exposure, and lack of clinical mindfulness led to a 10-day delay in diagnosis.

The clinical course of skin mucormycosis varies widely. It can appear either gradually or slowly progressive. It may be even fulminant resulting gangrene and hematogenous dissemination [5]. Hematogenous dissemination usually occurs in immunocompromised patients [1, 2, 7]. Our patient had an excellent clinical course without any evidence of local or systemic progression after surgery. Biopsy of the lesions and histopathology and culture studies are essential for diagnosis. Broad, large, and mostly nonseptate fungal hyphae are characteristic of Zygomycetes. Cultures are positive in around 50% of cases and as in our case; *Rhizopus* Spp. is one of the most common pathogen in positive specimens [2, 8]. Other species isolated from clinical specimens are *Absidia corymbifera*, *Apophysomyces elegans*, *Mucor* Spp., *Rhizomucor* Spp., *Cunninghamella bertholletiae*, and *Saksena vasiformis*.

The mainstays of the successful management of patients with mucormycosis are early diagnosis, treatment of underlying disorders, aggressive surgical debridement, and use of appropriate antifungal therapy [1, 2, 9]. 

Amphotericin B is the drug of choice for treatment of mucormycosis. Liposomal form allows the administration of higher doses (5–7 mg/Kg) with less nephrotoxicity [2]. Although high production costs limit the wide use of these drugs, especially for patients living in low income countries like our patient. The length of treatment is undefined and it is highly individualized. Near normalization of radiographic imaging, negative biopsy specimens and or cultures, and correction of underlying disorders are indicators for stopping antifungal therapy [10]. Amputation of the affected limb and no evidence of localized or systemic dissemination of infection more than one week after surgery were our reasons that this patient is a candidate for stopping amphotericin b. Extensive surgical debridement with a cutoff of uninfected tissue is mandatory. Amputation is usually unavoidable if the affected area is an extremity [2]. Delay in surgical debridement, until amphotericin b started to show its effect, was related to a better outcome in one case report [6].

The prognosis of cutaneous mucormycosis is better than the other clinical forms of the disease. However the mortality is still high, ranging from 4% (in localized form) to 94% (in disseminated disease). Delay in diagnosis and antifungal treatment, evidence of tissue infarction and thrombosis, and uncorrected underlying conditions raise the mortality [2, 11]. 

Amputation of the affected limb after 8 days of antifungal therapy led to a good outcome in our case, despite invasion of the fungal infection to the muscles, tendons, and vessels of the limb and delay in diagnosis. 

In summary, lessons needed to be learned from this case report are as follows. First, in a patient presenting with uncommon clinical findings, a common disease with an unusual presentation, and a rare disorder, both should be considered. Second, the existence of more than one fascinating factor in a patient can raise the possibility of an uncommon disorder. Accordingly, in the case accompanying diabetes mellitus, trauma, and skin contact with contaminated materials were associated with the development of skin mucormycosis.
